# Advances in Environmental Psychology

**DOI:** 10.3390/bs5030384

**Published:** 2015-09-09

**Authors:** Jack L. Nasar

**Affiliations:** Department of City and Regional Planning, Austin E. Knowlton School of Architecture, The Ohio State University, Knowlton Hall, 275 West Woodruff Ave. Columbus, OH 43210-1135, USA; E-Mail: nasar.1@osu.edu

When Plenum stopped publishing its edited series—*Human Behavior and Environment* and *Advances in Environment, Behavior and Design*—the field of environmental psychology suffered a loss. Scholars could go to one of the edited Plenum books to find state-of-the-art reviews on existing and emerging areas of research. When *Behavioral Sciences* approached me to guest edit a Special Issue on Advances in Environmental Psychology, I saw it as a chance to revive the lost resource. I checked with *Behavioral Sciences* to ensure that I would have complete control over the editing process (meaning that I would invite individuals to write reviews and those invited authors would not have any publication charges, that I would set deadlines, select reviewers, read and decide how to proceed based on the reviews, and if uninvited people submitted manuscripts, I would control the review process on their papers as well.

*Behavioral Sciences* agreed, I contacted potential authors, and the *Behavioral Sciences* website posted my call for papers, which stated: “Environmental psychology is an interdisciplinary and international field that views persons and their physical surroundings as interdependent. It uses social science methods to study those person–environment relations, and recognizes the value of a multi-level, multi-disciplinary, social-ecological approach to such questions. This Special Issue explores the connections between the environment (at different scales, ranging from a room to a city) and the range of human responses addressed in the field. These connections and related responses include, but are not limited to, environmental perception and cognition; environmental attitudes and appraisals; environmental stress, noise, and crowding; responses to disasters, settings, personal space, territoriality, and privacy; crime and fear of crime; behavioral change; home, neighborhood, work, and educational environments; and facility planning and evaluation. Articles appropriate for the Special Issue include historical perspectives, theoretical articles, and reviews of research in a topic area, or discussions of a program of empirical research in an area. Papers that examine the relations between humans and their surroundings with planning, design or policy implications would represent excellent fits. This Special Issue aims to explore the state of knowledge in the field and the application of that knowledge to creating better places for people.”

Each of the eight published manuscripts went through a rigorous review process (with two or more expert reviewers). As an urban planner/urban designer, I am interested in neighborhood and urban issues. Some papers touch on such issues, but others deal with smaller scale building and setting issues. Some center more on theory and others more on practical applications. The accepted papers include:

- Rollings, A. K., Wells, N. M. and Evans, G. 2015. Measuring neighborhood quality related to health. *Behav. Sci. 5*(2), 190–202.
- N. L. Mihavlov and D. D. Perkins. 2015. Local environmental grassroots activism: Contributions from environmental psychology, sociology and politics. *Behav. Sci., 5*(1), 121–153.
- Reybrouck, M. 2015. Music as environment: An ecological and biosemiotic approach. *Behav. Sci, 5*(1), 1–26.
- Stamps, A. A. 2014. Protocol for evaluating contextual design principles. *Behav. Sci., 4*(4), 448–470.
- Devlin, A. S. 2014. Wayfinding in healthcare facilities: contributions from environmental psychology. *Behav. Sci., 4*(4), 423–436.
- Berto, R. 2014. 2014. The role of nature in coping with psycho-physiological stress: a literature review on restorativeness. *Behav. Sci. 4*(4), 394–409.
- Heft, H., Hoch, J. Edmunds, T. and Weeks, J. 2014. Can the identity of a behavior setting be perceived through patterns of joint action? An investigation of place perception. *Behav. Sci., 4*(4), 371–393.
- Terzano, K. 2014. Commodification of transitioning ethnic enclaves. *Behav. Sci., 4*(4), 341–351.

## Appendix

### Authors

**Rita Berto**, Ph.D. in perception and psychophysics, is a psychologist specialized in environmental psychology. She was Visiting Research Associate in Psychology (Honorary) at the Washington University in St. Louis (MO), and Professor of Environmental Psychology and Cognitive Psychology at the University of Padova. Her research interests cover the perception of restorativeness from the life-span perspective, the effects of exposure to natural environment on cognition and biophilic architecture. Her biography was included in Who’s Who in the World 2007. She is author of a book on Environmental Stress.

**Ann Sloan Devlin** is the May Buckley Sadowski '19 Professor of Psychology at Connecticut College. She has published extensively on healthcare environments, in particular the role of the facility’s appearance in people’s judgments of expected care. Her current research includes perceived control, social support, and positive distractions as mediators of stress in healthcare settings. Another focus of her research is perceptions of psychotherapists’ offices. Devlin's 4th book (2015) Transforming the Doctor’s Office: Principles from Evidence-based Design focuses on healthcare environments and small outpatient settings, in particular. She is past Environmental Design Research Association board member and secretary. She is a Fellow of Division 34 of the American Psychological Association.

**Gary Evans** is an environmental and developmental psychologist interested in how the physical environment affects human health and well-being among children. His specific areas of expertise include the environment of childhood poverty, children's environments, cumulative risk and child development, environmental stressors, and the development of children's environmental attitudes and behaviors.

**Trent Edmunds** is a Graphic Designer in Information Technology Services at Denison University.

**Harry Heft** is Professor of Psychology at Denison University. His scholarly interests primarily concern topics in the related areas of environmental and ecological psychology. His book "Ecological Psychology in Context" (Taylor and Francis, 2001) elucidates the theoretical and philosophical foundations of ecological psychology and some of its connections to current work in cultural psychology.

**Justine E. Hoch** is presently a Ph.D. student in developmental psychology at New York University. She has served as the manager of a developmental psychology laboratory at The University of California Berkeley and received a B.S. degree in psychology from Denison University in 2012.

**Nikolay Mihaylov** is a doctoral candidate in Community Research and Action, Vanderbilt University, USA. He has degrees in Economics and Clinical Psychology, and is a certified group trainer. His work focuses on the exploration of the actions and processes that bring people together for a common cause, including school communities, social movements, and interactions of citizens with institutions. He is interested in both the local (community organizing) and societal (social movements) levels of collective action. For his dissertation, he is researching the successful movement against “fracking” (hydraulic fracturing energy development) in Bulgaria. He is currently teaching in Bulgaria and is a consultant to community and civic organizations working on grassroots democracy, environmental, and education issues.

**Jack L. Nasar (Editor)** is a Professor Emeritus in City and Regional Planning at Ohio State University. His scholarship centers on environmental perception, cognition and aesthetics with an emphasis on urban design. His book *The Evaluative Image of the City* (Sage, 1998) extends Lynch’s *Image of the City* from environmental cognition to environmental preferences.

**Douglas D. Perkins** is a community/environmental psychologist and Professor of Human and Organizational Development, Vanderbilt University, USA. He directs the Ph.D. Program in Community Research and Action and also teaches in the Community Development and Action M.Ed. Program and the Community Leadership and Development undergraduate track. He is former Chair of the Public Policy Committee of the Society for Community Research and Action. His collaborative and ecologically-oriented research addresses youth, organizational, and community development, housing, violence, crime, fear, social capital (participation, empowerment, neighboring, sense of community), and disorder in urban neighborhoods in America, Europe, China, and Africa.

**Mark Reybrouck** studied physical education, physical therapy and musicology. He is professor of music at the University of Leuven (Belgium). His major research is interdisciplinary with an attempt to bring together insights from the fields of psychology, biology, semiotics and music. His research agenda concerns listening strategies and musical sense-making with a major focus on musical semantics and biosemiotics as applied to music. At a theoretical level he is involved in foundational work about music cognition and perception, especially the biological roots of musical epistemology and the embodied enactive approach to dealing with music. He is also involved in empirical research on representational and meta-representational strategies in music-listening tasks.

**Kimberly Rollings** is an Assistant Professor in the School of Architecture at the University of Notre Dame. Trained as an architect, Dr. Rollings systematically assesses how design characteristics of the built and natural environments affect mental and physical health across multiple scales, especially among vulnerable populations. Additionally, she develops environmental assessment tools to quantify attributes of the physical environment related to health. Assessment tool results inform planning, design, and policy with the goal of establishing healthy, socially responsible, and sustainable buildings and cities.

**Arthur Stamps** received his Ph.D. from U.C. Berkeley in 1980. The work focused on statistics (done in the psychology department), philosophy (in the Graduate School of Business), and futures research (Department of City Planning). He has published over 100 articles and made seminal contributions on the topics of environmental simulation, scaling, scientific protocols for performing design review, demographic effects on environmental preferences, spatial permeability and enclosure, the mystery and prospect and refuge theories of environmental preference, contextual design, information theory, environmental preference and exploration, and peer review. He works at the Institute of Environmental Quality in San Francisco.

**Kathryn Terzano** is an Assistant Professor of Geography and Regional Planning at Westfield State University in Westfield, Massachusetts. Her research focuses on healthy and sustainable community design, including aspects of neighborhood satisfaction, non-motorized transportation, and social equity. She earned her doctorate in City and Regional Planning from The Ohio State University.

**Jillian J. Weeks** is presently a Ph.D. student in neuroscience at the University of Pittsburgh. She received a B.S. degree in psychology from Denison University in 2015.

**Nancy M. Wells** is an Associate Professor in the Department of Design and Environmental Analysis at Cornell University. As an environmental psychologist, Dr. Wells studies the effects of the built and natural environment on human health and well-being. Her work focuses on housing quality and mental health; nature and cognitive functioning; and the influences of the environment on physical activity and diet.

